# Self-organizing actin waves that simulate phagocytic cup structures

**DOI:** 10.1186/1757-5036-3-7

**Published:** 2010-03-18

**Authors:** Günther Gerisch

**Affiliations:** 1 Max-Planck-Institut für Biochemie, Am Klopferspitz 18, D-82152 Martinsried, Germany

## Abstract

This report deals with actin waves that are spontaneously generated on the planar, substrate-attached surface of *Dictyostelium *cells. These waves have the following characteristics. (1) They are circular structures of varying shape, capable of changing the direction of propagation. (2) The waves propagate by treadmilling with a recovery of actin incorporation after photobleaching of less than 10 seconds. (3) The waves are associated with actin-binding proteins in an ordered 3-dimensional organization: with myosin-IB at the front and close to the membrane, the Arp2/3 complex throughout the wave, and coronin at the cytoplasmic face and back of the wave. Coronin is a marker of disassembling actin structures. (4) The waves separate two areas of the cell cortex that differ in actin structure and phosphoinositide composition of the membrane. The waves arise at the border of membrane areas rich in phosphatidylinositol (3,4,5) trisphosphate (PIP3). The inhibition of PIP3 synthesis reversibly inhibits wave formation. (5) The actin wave and PIP3 patterns resemble 2-dimensional projections of phagocytic cups, suggesting that they are involved in the scanning of surfaces for particles to be taken up.

**PACS Codes**: 87.16.Ln, 87.19.lp, 89.75.Fb

## 1. Introduction

This article is an overview of work supported by the Deutsche Forschungsgemeinschaft in the Priority Program "Optical analysis of the structure and dynamics of supramolecular biological complexes". Subject of our studies has been the self-organization of actin into propagating waves. I review published results on the dynamics and molecular composition of these waves and will elaborate on some connotations that are not detailed elsewhere.

The starting question we addressed was: how are actin structures organized de novo in cells that have been depleted of polymerized actin? To inhibit actin polymerization, we have used latrunculinA (LatA). In living cells, this scavenger of actin monomers results in the depolymerization of actin filaments at a rate determined by their turnover. The disassembly and re-assembly of actin structures has been monitored in *Dictyostelium *cells using a GFP- tagged construct (LimEΔ) that proved to be an appropriate label for recording the dynamics of filamentous actin structures in these cells [1]. The actin structures rapidly turn over, resulting in microscopically detectable breakdown of the actin network in the cell cortex within less than 20 seconds after LatA addition [2].

The removal of LatA enables the cells to regain normal actin organization and cell motility within less than an hour. Of interest here is the intermediate state of excessive wave formation before normal cell motility will recover. This state is preceded by the formation of mobile actin patches [3]. The burst of patches at the onset of actin polymerization is mostly due to the fact that clathrin-dependent endocytosis requires actin to polymerize in order for vesicles to be budded off. Therefore, many clathrin-coated pits are arrested at the plasma membrane as long as LatA is present. Accordingly, the first actin structures seen within about 5 minutes after removal of the drug are small, clathrin-induced patches. Later on, actin waves are generated in a spatial relationship to the synthesis of phosphatidylinositol-(3,4,5) trisphosphate (PIP3) in the plasma membrane. We have used the time window of excess formation of actin waves for the experimental analysis of their structure and mode of propagation [4].

## 2. Materials and methods

Cells of *Dictyostelium discoideum *strain AX2-214 were transfected with expression vectors encoding GFP- or mRFP-fusion proteins [4] and subjected to imaging at 23 ± 2°C in 17 mM Na/K-phosphate buffer, pH 6.0 (PB) according to [3]. For the recovery of actin polymerization, cells were pretreated for about 15 minutes with 5 μM latrunculin A (Invitrogen). Subsequently, the drug was replaced with PB. To inhibit PI3-kinases, a stock solution of 30 μM LY-294002 (Sigma) in DMSO was diluted to 50 μM in PB and added to the cells during the wave-forming stage of recovery. For the imaging of clathrin-coated structures in relation to reversible inhibition of wave formation, a Zeiss LSM 510 equipped with a 63x/1.4 oil apochromate objective was used. TIRF microscopy was applied to actin structures according to [5]. Spinning-disc confocal microscopy was performed as detailed in [4].

For phagocytosis experiments, *D. discoideum *cells, double-labeled as for wave formation, were exposed to living *Saccharomyces cerevisiae *[6]. In the phagocytic cups formed around these large particles, the accumulation of GFP-tagged proteins relative to filamentous actin labeled with mRFP-LimEΔ was recorded using the Zeiss LSM 510 confocal microscope.

## 3. Results

### 3.1. Structure and dynamics of self-organizing actin waves

**Figure 1:**
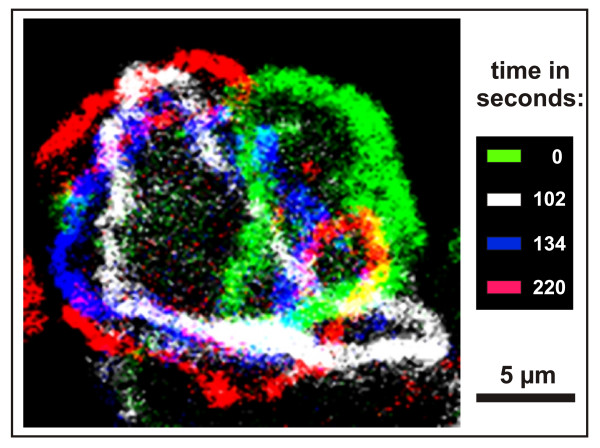
**Shape dynamics of actin waves viewed on a substrate-attached cell surface**. Wave images have been recorded from a single cell of *Dictyostelium discoideum *at the indicated times by spinning-disc confocal microscopy. During propagation of the wave, the cell showed negligible net movement. The images are color-coded and superimposed on top of each other. Shape changes within a period of less than 2 minutes are best recognized by comparing the green and white images. Waves can fuse or they may split into two as in the red image at the end of the recorded sequence. The images are taken from Figure 1 in [5].

**Figure 2:**
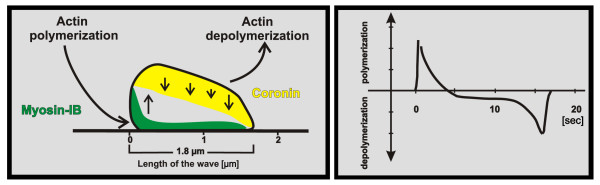
**Spatial organization of actin waves and the temporal pattern of actin polymerization and depolymerization**. **Left panel**: schematic cross-section through an actin wave showing the region at the front and close to the membrane where myosin-IB is enriched (green) and the sloping roof of the wave to which coronin is recruited (yellow). Vertical arrows indicate up and down regulation of actin polymerization. Data suggest two gradients of actin polymerization, one falling from the substrate-attached membrane to the top of the wave, the other from its front to the tail. **Right panel**: translation of the spatial profile into a temporal sequence of actin net polymerization and depolymerization. The profile illustrates the sequence of changes that occur when a wave passes over a point on the membrane. The data published in [4] suggest that an initial phase of high-rate actin polymerization turns into a longer phase of depolymerization.

The actin waves studied here are typically circular structures of varying shape (Figure 1). They change their shape by propagating at the substrate-attached surface of *Dictyostelium *cells with velocities of about 100 nm per second [4]. The waves propagate in a treadmilling mode, with net polymerization of actin at their front and net depolymerization at the back. Even when the waves keep their overall shape during propagation, their constituting proteins are exchanged: after photobleaching, half-maximal fluorescence recovery of actin has been observed within 4 s, and of myosin-IB within 2 s [4]. The Arp2/3 complex is distributed throughout the wave, indicating that the entire wave structure is dominated by a dense fabric of branched actin filaments. Three-dimensional scanning of protein distributions using spinning disc confocal microscopy revealed distinct patterns for myosin-IB and coronin in cross-sections through a wave. Myosin-IB, a single-headed motor protein capable of binding to the plasma membrane, is enriched at the front of the wave and at the substrate-attached area of the membrane. Coronin occupies the sloping roof of the wave at its cytoplasmic face (Figure 2, left panel). Assuming that membrane-anchored myosin-IB clusters are sites of active actin polymerization whereas coronin is recruited to sites of depolymerization, two gradients of actin polymerization can be proposed: one gradient declining from the front to the back of a wave, the other from the substrate-attached membrane to the roof of the wave (Figure 2, right panel).

### 3.2. Actin waves are confined by different states of actin organization and by membrane areas of different phosphoinositide composition

**Figure 3:**
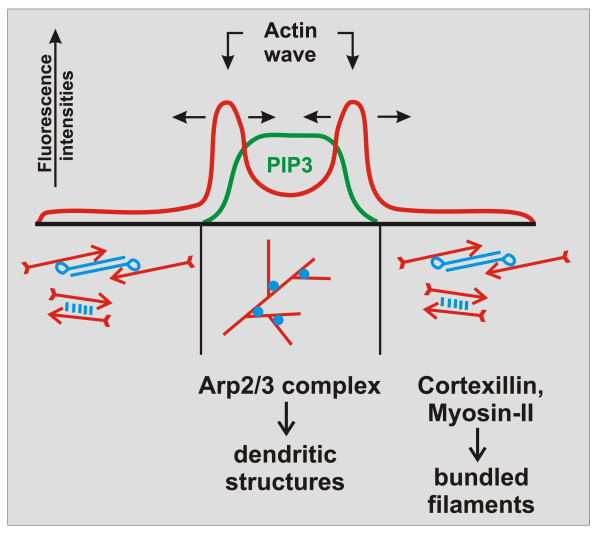
**Spatial relationship of actin organization to the presence or absence of PIP3 in the underlying membrane**. The diagram represents a line scan through an actin wave pattern on a planar glass surface. On top, the relationship of actin- and PIP3-labels is shown. The fluorescence intensity of actin (red) peaks at the position of the wave formed at the border of a PIP3-rich area of the cell membrane (green). Horizontal arrows point into the changing directions of wave propagation. On the bottom, actin structures are shown to differ between the PIP3-rich area circumscribed by the wave and the external area depleted of PIP3. The Arp2/3 complex known to nucleate branches of actin filaments dominates in the PIP3-rich area, while proteins associated with anti-parallel bundles of filaments are prevailing in the external area. This diagram summarizes data published in [5] and [7].

In order to probe for actin structures in the cortex of wave-forming cells, we used dual-color fluorescence labeling of proteins that associate with different arrangements of actin filaments [5]. The area circumscribed by a wave turned out to be enriched in the Arp2/3 complex, indicating a dominance of dense dendritic actin structures in this inner area. In contrast, cortexillin I, a protein that bundles actin filaments in anti-parallel direction, prevails in the external area, together with myosin-II, a conventional myosin that forms bipolar filaments (Figure 3). Since the waves can change the direction of propagation, the two areas reciprocally increase or decrease in size.

Labeling of phosphoinositides revealed that actin waves are formed at the boundary of a membrane area rich in PIP3 [7]. Enrichment of the Arp2/3 complex in this inner area suggests that PIP3 directs the assembly of actin into dendritic structures, while in the external area depleted of PIP3 a basal network of bundled actin filaments is dominating. The reciprocal expansion and shrinkage of these areas demonstrates a connection between PIP3 regulation and conversion of one state of actin organization into the other (Figure 3). A linkage of actin waves to PIP3 in the membrane has previously been found under other conditions [8]. This linkage is in line with the control of actin polymerization through a positive feedback loop involving PIP3 and Ras [9]. However, under our conditions the actin waves do not coincide with regions of strongest PIP3 accumulation but with zones where PIP3 forms the steepest gradients in the membrane. The control mechanisms underlying this complex behavior remain to be elucidated.

**Figure 4:**
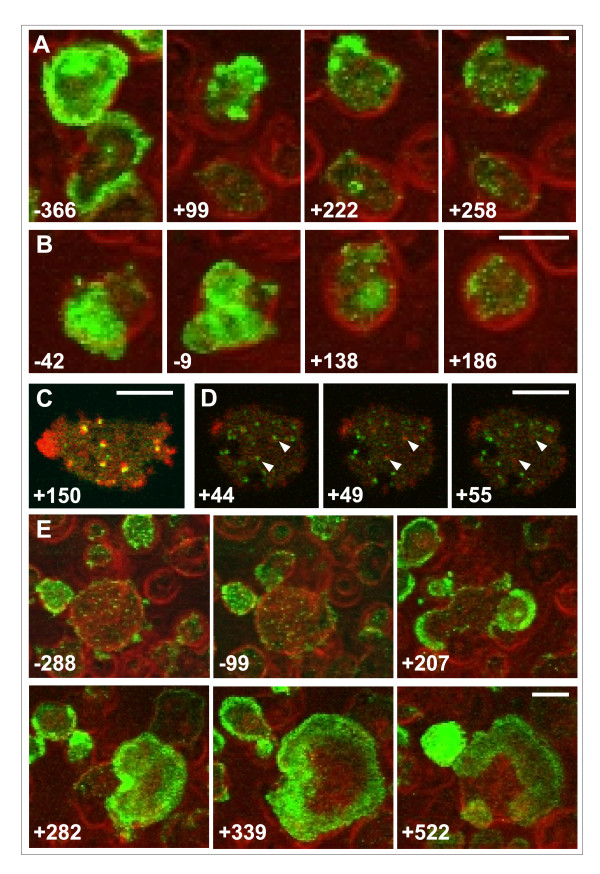
**Reversible suppression of actin wave formation by the PI3-kinase inhibitor LY-294002**. Cells recovering from LatA treatment were allowed to form waves before 50 μM LY-294002 were added. In (A to D) time is indicated in seconds before and after addition of the inhibitor (t = 0), in (E) before and after its removal. In (A, B, and E) the LimEΔ-GFP label for actin (green) is superimposed on phase-contrast images showing cell shape (red). In (C and D) cells are double-labeled with mRFP-LimEΔ for actin (red) and GFP-clathrin light chains for clathrin-coated structures (green). **A**, **B**, actin wave formation is suppressed by LY-294002 while small actin patches are persisting. **C**, co-localization of the actin and clathrin labels in six patches at the substrate-attached surface of an LY-treated cell. **D**, clathrin and actin dynamics in an LY-treated cell. Arrowheads point to clathrin-coated structures (44 s) that recruit actin thus turning from green to red (49 s) and subsequently disappear from the membrane (55 s). **E**, recovery of wave formation in a big cell after the removal of Ly-294002. Bars, 10 μm in (A, B, and E); 5 μm in (C and D).

If the formation of actin waves relies on a pattern of PIP3 in the underlying membrane, wave formation should be suppressed by the PI3-kinase inhibitor LY-294002 (Figure 4). In fact, the formation of actin waves is sensitive to 50 μM LY-294002, a concentration which reduces PI3-kinase activity in vivo [10]. Upon addition of the inhibitor, waves disappear within 2 minutes (Figure 4A, B). Only short actin-rich protrusions are transiently formed, and numerous actin patches decorate the substrate-attached membrane of the LY-treated cells. To find out whether these patches are rudimentary actin waves or distinct structures, we have co-expressed GFP-tagged clathrin light-chains together with the mRFP-tagged actin label, since actin patches of similar size are known to be involved in clathrin-dependent endocytosis [11]. The two labels coincided, indicating that, at the concentration used, LY-294002 neither prevents clathrin-coated structures from inducing actin polymerization (Figure 4C) nor from becoming internalized (Figure 4D).

The suppression of actin waves is readily reversible. Within 3 minutes after removal of the inhibitor, profuse formation of actin waves recovered (Figure 4E). In conclusion, the self-organization of actin waves is distinguished by its strong dependence on PIP3 from the activity of clathrin-induced actin patches, such that clathrin-dependent endocytosis can be separated from wave formation by the inhibition of PIP3 synthesis.

### 3.3. Protein and PIP3 patterns in phagocytic cups correspond to the patterns in wave-forming cells

Like other phagocytes, *Dictyostelium *cells interact with adhesive surfaces in two ways: on a planar surface they spread and migrate, on a convex surface a circular protrusion is induced to try and engulf a particle. By progression of its rim along the particle surface, this phagocytic cup envelops and eventually encloses the particle by separation of the phagosome membrane from the plasma membrane. Filamentous actin accumulates between the outer and inner leaflet of the cup membrane and is most strongly enriched at the rim of the cup, the site of its protrusion. The leaflet of the cup membrane that is in contact with the particle becomes rich in PIP3 [10,12].

**Figure 5:**
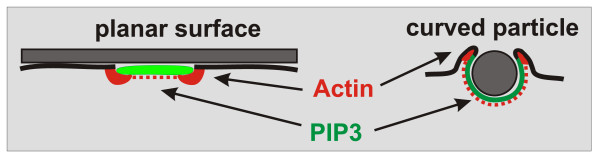
**Comparison of actin-PIP3 patterns on a planar surface and in phagocytic cups**. **Left**: autonomous actin waves (red) circumscribe a PIP3-rich area of the substrate-attached cell membrane (green). **Right**: similarly, an actin ring at the rim of a phagocytic cup surrounds the PIP3-rich membrane area invaginated in contact with a particle. Dotted red lines indicate accumulation of filamentous actin on top of PIP3-enriched membrane areas. This diagram is based on data reported in [7].

With regard to the biological function of the actin waves generated on a planar surface, it is appealing to consider them as devices to search for particles to be taken up. In fact, the state of excessive wave formation in cells recovering from actin depolymerization is characterized by a high propensity for taking up particles with a minimum of net movement of the cells on a planar surface [7]. If the particle-induced actin and PIP3 patterns in a phagocytic cup are projected onto a plane, they coincide with the spontaneously generated patterns in wave-forming cells (Figure 5). More complicated patterns are observed in cups formed around long rod-shaped particles. These patterns correspond to toroid-like actin-wave and PIP3 patterns that are formed in large cells on a planar surface [7].

**Figure 6:**
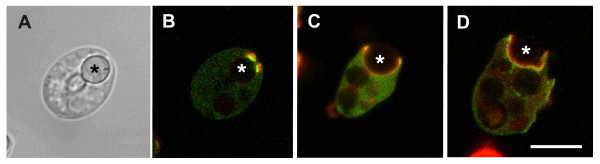
**Arrangement of actin-associated proteins in phagocytic cups**. During the uptake of yeast particles, cells were imaged to localize GFP-tagged myosin-IB, Arp2/3, or coronin (green) in combination with filamentous actin (red). Centers of the particles are indicated by asterisks. **A**, bright-field image of a late phagocytosis stage showing a particle being engulfed. **B**, double-fluorescence recording of the same cell showing GFP-myosin-IB enriched at the border of the cup close to the plasma membrane. **C**, uptake of a particle by a cell expressing GFP-Arp3, indicating coincident localization of the Arp2/3 complex and the labeled actin. **D**, the phagocytic cup in a cell expressing GFP-coronin shows coronin remote from the membrane at the interface between the actin layer and the cytoplasmic space. The progressing edge of the cup is free of coronin. Bar, 10 μm.

To underscore the similar organization of spontaneously generated wave patterns and particle-induced phagocytic cups, we have compared the characteristic localization of three actin-associated proteins in cups with their localization in waves as reported in [4]. In the cups, myosin-IB is enriched close to the membrane at the very border of the protruding cup (Figure 6). The Arp2/3 complex is distributed throughout the actin layer of the cup. Coronin is again recruited to regions where actin is supposed to depolymerize: at the sides and the bottom of the cup, specifically at the boundary between actin layer and cytoplasmic space remote from the membrane (Figure 2).

For the optical analysis of the pattern dynamics, the planar arrangement of pattern elements in wave-forming cells has an advantage; phase transitions in the cell cortex can be recorded by TIRF microscopy at high temporal and spatial resolution. At each point of the substrate-attached surface of a wave-forming cell the actin structure undergoes transitions from one state to the other. The temporal sequences of these transitions are variable; they may be irregular or ordered into patterns with periods of about 5 minutes [5].

## 4. Discussion

### 4.1. Relation of wave propagation in Dictyostelium to other actin-based cell functions

The actin waves in *Dictyostelium *cells are macromolecular complexes with a defined 3-dimensional architecture. While propagating, the waves continuously turn over their constituents. The organization of these waves shows that self-sustained actin structures can be generated without a membrane acting as a scaffold and site of activating factors in front of these waves. The most relevant feature of the actin waves is their resemblance to functional actin structures that promote the extension of a phagocytic cup (Figures 5 and 6). Similar patterns of actin-associated proteins are assembled during leading edge protrusion, phagosome rocketing [13], cell spreading or clathrin-dependent membrane internalization [11]. In all these cases is actin associated with the Arp2/3 complex. A single-headed myosin, myosin-IB, is enriched at the front of the dynamic actin structures close to the underlying membrane, and coronin is always recruited to regions of actin disassembly. Characteristic of the actin waves is their localization at the boundary between PIP3 rich and depleted areas of the plasma membrane. This localization coincides with the boundary between two states of actin organization, one resembling the front region of a freely migrating or chemotaxing cell, the other corresponding to the tail region rich in filamentous myosin-II [5].

The actin waves studied by us differ in their organization, dynamics and function from actin-based waves of Hem-1, a constituent of the Arp2/3-activating WAVE complex [14,15]. Hem-1 waves propagate in neutrophils at intervals in one direction and are oriented by chemoattractant, whereas the actin waves in *Dictyostelium *are typically circular and related to the phagocytic activity of these cells.

### 4.2. Actin waves are comparable to trigger waves in bistable systems

The finding that the actin waves studied in *Dictyostelium *cells are confined at their front and back by two different states of actin organization is pertinent to the question of whether or not the actin waves are periodic structures formed in an excitable medium that, after a wave has passed, returns to its resting state ready to form a new wave. Different from these structures, the actin waves generated in *Dictyostelium *cells are confined at their front and back by two distinct states of actin organization. In this respect, the wave-forming actin cortex resembles those pattern-generating reaction-diffusion systems in which far from equilibrium bistability is established. Waves that separate the two phases in such systems are known as "trigger waves" [16]. A model specifying conditions under which bistability will arise in the actin cortex is provided by Carsten Beta in this issue [Beta C: A bistable model of actin dynamics. PMC Biophysics, submitted].

In summary, actin waves separate two large areas of the cell cortex that are distinguished by their actin structure and by the PIP3 content of the membrane. Actin structure and membrane composition of these areas are reciprocally interconverted when the actin waves propagate into one or the other direction. Since the wave patterns are formed in contact with a planar glass surface, they provide an opportunity to analyze phase transitions in the actin system under superior optical conditions using TIRF microscopy and to correlate these transitions with the regulation of phosphatidylinositides in the membrane.

## References

[B1] Schneider N Weber I Faix J Prassler J Müller-Taubenberger A Köhler J Burghardt E Gerisch G Marriott G Cell Motil Cytoskeleton 2003 56 130 139 10.1002/cm.10139 14506710

[B2] Diez S Gerisch G Anderson K Müller-Taubenberger A Bretschneider T Proc Natl Acad Sci USA 2005 102 7601 7606 10.1073/pnas.0408546102 15894626 PMC1140407

[B3] Gerisch G Bretschneider T Müller-Taubenberger A Simmeth E Ecke M Diez S Anderson K Biophys J 2004 87 3493 3503 10.1529/biophysj.104.047589 15347592 PMC1304815

[B4] Bretschneider T Anderson K Ecke M Müller-Taubenberger A Schroth-Diez B Ishikawa-Ankerhold HC Gerisch G Biophys J 2009 96 2888 2900 10.1016/j.bpj.2008.12.3942 19348770 PMC3325131

[B5] Schroth-Diez B Gerwig S Ecke M Hegerl R Diez S Gerisch G HFSP J 2009 3 412 427 10.2976/1.3239407 PMC2839813 20514132

[B6] Clarke M Maddera L Engel U Gerisch G PLoS ONE 2010 5 e8585 1 14 10.1371/journal.pone.0008585 PMC2796722 20052281

[B7] Gerisch G Ecke M Schroth-Diez B Gerwig S Engel U Maddera L Clarke M Cell Adh Migr 2009 3 373 382 19855162 10.4161/cam.3.4.9708 PMC2802751

[B8] Asano Y Nagasaki A Uyeda TQP Cell Motil Cytoskeleton 2008 65 923 934 10.1002/cm.20314 18814278

[B9] Sasaki AT Janetopoulos C Lee S Charest PG Takeda K Sundheimer LW Meili R Devreotes PN Firtel RA J Cell Biol 2007 178 85 191 10.1083/jcb.200611138 17635933 PMC2064438

[B10] Dormann D Weijer G Dowler S Weijer CJ J Cell Sci 2004 117 6497 6509 10.1242/jcs.01579 15572406

[B11] Heinrich D Youssef S Schroth-Diez B Engel U Aydin D Blümmel J Spatz JP Gerisch G Cell Adh Migr 2008 2 58 68 19262103 10.4161/cam.2.2.6190 PMC2634985

[B12] Loovers HM Kortholt A de Groote H Whitty L Nussbaum RL van Haastert PJM Traffic 2007 8 618 628 10.1111/j.1600-0854.2007.00546.x 17343681

[B13] Clarke M Müller-Taubenberger A Anderson KI Engel U Gerisch G Mol Biol Cell 2006 17 4866 4875 10.1091/mbc.E06-04-0365 16971511 PMC1635377

[B14] Weiner OD Marganski WA Wu LF Altschuler SJ Kirschner MW PLoS Biol 2007 5 2053 2063 10.1371/journal.pbio.0050221 PMC1945041 17696648

[B15] Millius A Dandekar SN Houk AR Weiner OD Curr Biol 2009 19 253 259 10.1016/j.cub.2008.12.044 19200726 PMC2705202

[B16] Mikhailov AS Foundation of Synergetics I 1994 Springer: Berlin, Heidelberg, New York 15 32

